# Probiotics for Preventing Ventilator-Associated Pneumonia: A Systematic Review and Meta-Analysis of High-Quality Randomized Controlled Trials

**DOI:** 10.1371/journal.pone.0083934

**Published:** 2013-12-18

**Authors:** Jie Wang, Kai-xiong Liu, Felinda Ariani, Li-li Tao, Jing Zhang, Jie-Ming Qu

**Affiliations:** 1 Department of Pulmonary Medicine, Huadong Hospital Affiliated to Fudan University, Shanghai, China; 2 Department of Pulmonary Medicine, The First Affiliated Hospital of Fujian Medical University, Fuzhou, Fujian Province, China; 3 Department of Pulmonary Medicine, Zhongshan Hospital Affiliated to Fudan University, Shanghai, China; D'or Institute of Research and Education, Brazil

## Abstract

**Background:**

Ventilator-associated pneumonia (VAP) is considered to be a worldwide issue along with the development of supportive ventilation. The preventing strategy is of great importance for its poor prognostic and difficulties in treatment. Probiotics have been advocated as one of the possible preventive measures. We conducted a systematic review and meta-analysis to explore the potential benefits of probiotics.

**Methods:**

The databases, Web of science, PubMed, Ovid and Cochrane lib were searched for randomized controlled trials (RCTs) publications that compared the effectiveness of probiotics with placebo in the prevention of VAP. The incidence of VAP was considered as the primary endpoint, mortality, length of stay in intensive care units (ICUs), etiology of the infections were considered as secondary endpoints.

**Results:**

A total of 844 patients from 5 trials were subjected to meta-analysis. Probiotics did not significantly decrease the incidence of VAP (RR 0.94, 95%CI 0.85-1.04, p=0.22), however, the administration of probiotics reduced the risk of VAP caused by Pseudomonas aeruginosa (P. aeruginosa) (RR 0.30, 95%CI 0.11-0.91, P=0.03). It failed to affect any other endpoints.

**Conclusion:**

Probiotic prophylaxis of ventilator-associated pneumonia remained inconclusive and it failed to improve the prognosis of general mechanically ventilated patients. It was noteworthy that infections caused by P. aeruginosa was reduced by administration of probiotics. In further, it is recommended that advanced studies should exploit transformation in pathogenic microorganisms owing to administration of probiotics as well as the specific population.

## Introduction

Ventilator-associated pneumonia (VAP) remains to be one of the most prevalent and serious complications during ventilation. According to the available data, the incidence ranges from 6 to 52 per 100 cases, crude rates of VAP are usually 1 to 3% per day of intubation and mechanical ventilation. It is highlighted that VAP adds the burden of intensive care units (ICUs). Under the circumstances of ICUs, VAP accounts for 25% of all ICU infections and occurs in 9% to 27% of all intubated patients. The risk increases with prolonged ventilation duration, so does the mortality[1]. Intubation process itself contributes to the risk of infection and nearly 90% episodes of HAP in ICU occur during mechanical ventilation[1–4].

The endogenous flora play an important role in the development of VAP, the abnormal colonization and translocation of potential pathogenic microorganism (PPM) is believed to be the main pathogenesis. Microaspiration of oropharyngeal secretions contaminated by endogenous flora around the endotracheal tube cuff is the major route for microbial invasion. The stomach and sinuses are postulated as the potential reservoir of certain bacterial colonizing the oropharynx [5]. Colonization of the endotracheal tube with bacteria encased in biofilm results in embolization into the alveoli during suctioning or bronchoscopy while inhalation of pathogens from contaminated aerosols, and direct inoculation are less common. The most frequent medical conditions contributing to VAP episodes include intubation procedure, use of antibiotics, stressful situation, chronic respiratory diseases, gastroesophageal reflux diseases, ulcer prophylaxis, immunosuppression[1]. 

Numerous studies have exploited into the preventing strategies. The pharmacologic strategies aiming at eliminating the potential pathogenic microorganisms (PPM) are more potent than nonpharmacologic approaches that focus on strengthening the aseptic processing, altering posture, avoidance use of PPI, etc. Notably, it has been established that prophylactic use of antibiotics presents to be an effective preventive strategy against VAP [6–8], for example, selective digestive decontamination (SDD) and selective oropharyngeal decontamination (SOD) [6–8]. Unfortunately, the administration of antibiotics gains no benefit on the patients clinical outcomes since an increase in antibiotic resistance [9], but also places heavy burden on medical system.

On the ground of that, there raised an interest in the microecological preparations for attenuating or prevention of infectious diseases. Defined by WHO, probiotics are live microorganisms which confer a health benefit on the host when administered in adequate amounts, while prebiotics are poorly absorbed dietary oligosaccharides and synbiotics combines probiotics with prebiotics. They have gained reputation for effectiveness and safety in a variety of gastrointestinal illness[10]. The proposed mechanisms are complicated, including suppression or displacement of pathogenic bacteria, enhancement of innate immunity, and promotion of epithelial barrier function [11,12]. Earlier in 2005, probiotics was primarly used as potential adjunctive treatment for VAP prevention [13] in a randomized controlled trial (RCT) conducted in ventilated patients in ICU surroundings. The probiotics group surpassed the placebo group in maintaining oropharyngeal flora balance, eliminating the potential pathogenic microorganisms (PPM) in oral cavity and gastric juice. Though the result was not statistically significant, the non-antibiotic therapy was so intriguing that there arose a surge of studies exploring the effect of microecological preparations on critically ill patients. The effect of probiotics on VAP prevention has been controversial since the available high-quality RCTs were limited and yielded inconsistent results [14–22]. Then following meta-analysis [23–25] tried but in vain to establish a definite relationship between probiotics and VAP prevention. 

Therefore, we performed a systematic literature review and meta-analysis of high-quality randomized controlled trials to investigate the effect of probiotics in VAP prevention as the primary endpoint, mortality, length of stay in the ICU and in hospital, and responsible pathogen as secondary endpoints.

## Materials and Methods

### 2.1: Data sources

Two authors independently searched the databases including Web of science, Pubmed, Cochrane Lib, and OVID to screen for clinical trials published from January 2000 to April 2013. The searching phrases including “ventilator-associated pneumonia” or ” nosocomial pneumonia” or ”infections” and “prebiotics” or “probiotics” or “synbiotics” or “microecological preparations” or “lactobacillus” or “Bifidobacterium”. We also obtained data from the proceedings of major relevant conferences, trial databases, the reference lists of identified trials, and major reviews.

### 2.2: Study selection and appraisal

Two reviewers (JW and KXL) independently evaluated the eligibility of the identified publications after the literature searches. We included RCTs studies comparing probiotics with placebo treatment in adult patients undergoing MV and reporting on incidence of VAP. Therefore those using SDD as controlled group were excluded. The methodological quality of each trial was evaluated by the Jadad score[26]. Two reviewers (JW and KXL) independently appraised the quality of the studies and considered it of high quality if the score was ≥ 3.

### 2.3: Data Extraction

The traits of study: author, year of publication, location, study design, number of evaluated participants, population trait, diagnostic criteria of VAP.

The intervention: the detailed probiotics preparations used, dose, way of administration, the duration of treatment. Here the probiotics were referred to microecological preparations containing the composition of probiotics.

Endpoints: incidence of VAP, mortality, the duration of MV, length of stay in hospital, length of stay in ICU, the pathogens responsible for VAP. 

### 2.4: Data Analysis and Statistical Methods

STATA (STATA version 419.12.0.866; StataCorp LP) was used to perform statistical analyses. The presence of heterogeneity among trials was assessed by using the chi-square test (p <0.05 denoted statistical significance in the analysis of heterogeneity), whereas the extent of inconsistency was assessed using I^2^statistic. Publication bias was analyzed by drawing funnel plot method. Continuous variables were analyzed using weighted inverse variance and 95% confidence intervals (CIs) [27]. Pooled relative risks (RRs) and 95% CIs for categorical variables were calculated by implementing Mantel-Haenszel fixed effect model [28] or the DerSimonian-Laird random effects model [29].

## Results

### 3.1: RCTs included in the meta-analysis

**Figure 1:**
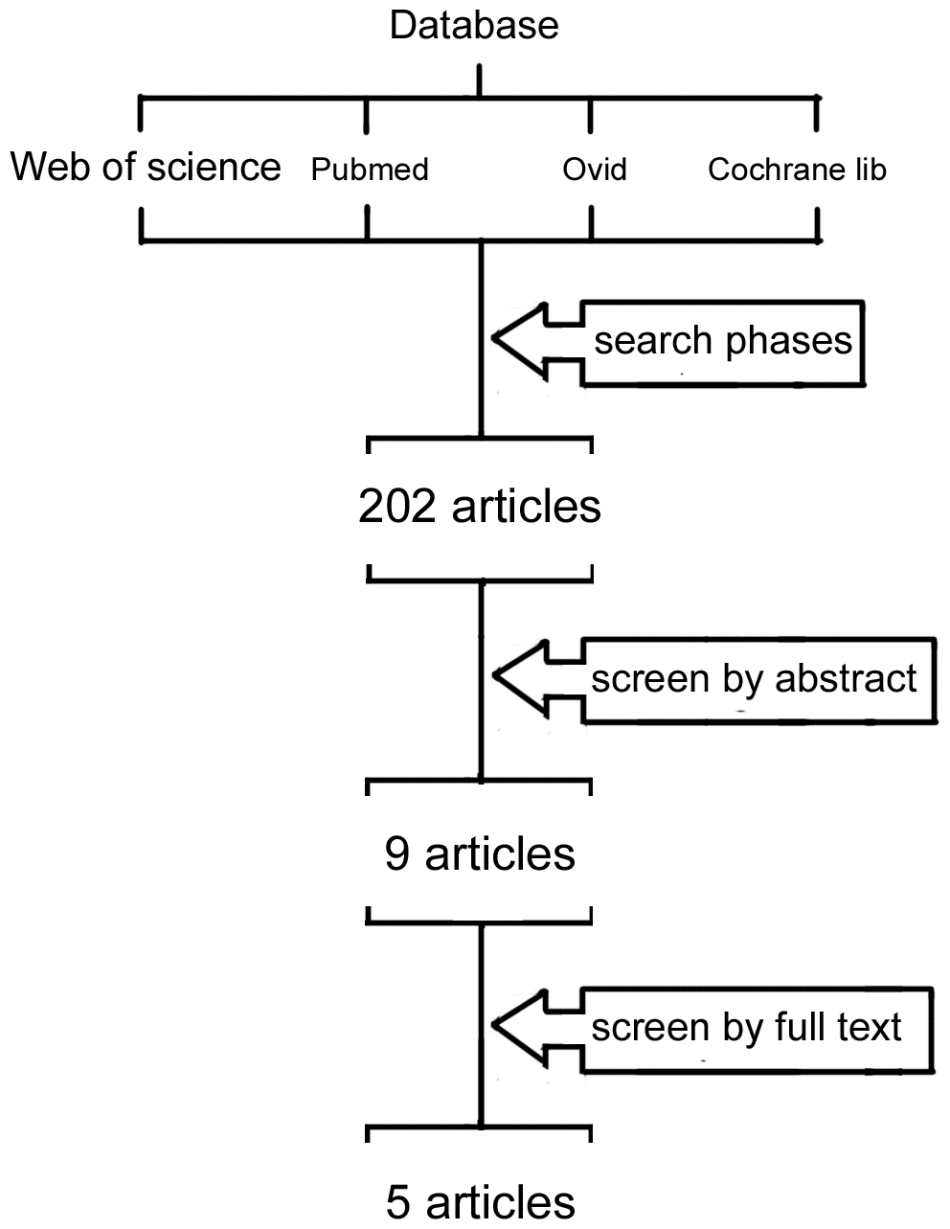
Selection process for RCTs included in the meta-analysis.

The initial literature research yield a number of 202 articles in total, most of which were excluded based on the titles and abstracts either for specifying in other infections or the study method. Nine articles remained eligible before advanced evaluation by full text reading. Two [18,22] of them chosen chlorhexidine (CHX) and selective digestive decontamination (SDD) as controlled group, which might overestimate the effect of placebo. Two articles [16,21] were consecutive reports of same RCT conducted in surgical ICUs and the summative one was adopted. The other study [20] failed to highlight on the VAP incidence. Thus, five studies [14–17,19] were eventually included in the meta-analysis. Figure 1 presented the selection process. 

### 3.2: Characteristics of the Selected Studies

In Table 1 we summarized the characteristics of the five RCTs[14–17,19] included in the current analysis. The sample ranged from 72 to 259 and the mean sample size was 168.8. “Adult” and “ventilator support” were necessary to enrolled studies, while different ventilated time was permitted. All studies were published after 2008 and with a Jadad Score of more than 4 points except for one article. Two RCTs [15,19] emphasized the need for quantitative cultures while the remaining required qualitative results. 

**Table 1:** Characteristics of RCTs Included in the Meta-analysis.

Study/year	Morrow et al/2010	Barraud et al/2010	GB et al/2009	David et al/2009/	Forestier et al/2008
Location	one university based hos	(-)	multi-center, teaching and military hos	one tertiary care university hos	one teaching hos
No.(pro/con)	138(68/70)	167(87/80)	72(36/36)	259(130/129)	208(102/106)
Including criteria	>19 y; undergoing intubation and MV	Intubated and under MV<2 days	Multiple organ injuries; urgent intubation and MV	Require MV>48h and with no contraindications to EN	>18 y; stay>48 h and a NT
Jaded score	5	4	3	5	5
Microecological preparation					
Species	L. rhamnosus GG	Multispecies probiotic^a^	Synbiotic 2000FORTE^b^	Synbiotic 2000FORTE^b^	L. casei rhamnosus
Doses(CFU/day)	4*10 ^9^	2* 10^10^	10^11^	2*10^11^	2*10 ^9^
Administration	NT or oropharynx tube	NT	NT or gastrostomy	NT	NT or orally
Length of treatment	Entire period of MV	Entire period of MV	15 d after admission	Entire period of MV; <28d	From the 3rd day after admission to discharge
Controlled group	placebo	placebo	placebo	placebo	placebo
Definition of VAP	CRI + 2 signs: 1.T>38.5°C or <35.0°C) 2.WBC>10,000/mm^3^ or <3,000/mm^3^ 3.purulent sputum	CRI + 1 sign: 1.purulent tracheal secretions 2.T>38.3°C 3.WBC>10,000/mm^3^ 4.positive quantitative cultures	All of the following: 1.new or persistent consolidation in lung X-ray 2.purulent TBS 3.CPIS>6	CRI + 2 signs: 1.T>38.3°C 2.WBC>12,000/mm^3^ or <4,000/mm^3^ 3.Purulent secretions	CRI+ positive quantitative cultures + 1 sign: 1.purulent sputum 2.T>38.5°C 3.positive blood culture 4.mini-BAL with more than 5% cells with intracellular bacteria.

a: mainly L. rhamnosus GG, but also L. casei, L. acidophilus, and Bifidobacterium bifidum

b: a combination of 10^11^ colony-forming units (CFU) of each one of the following LAB: P. pentoseceus, L. mesenteroides, L. paracasei, and L. plantarum and inulin, betaglucan, pectin, and resistant starch as bioactive fibers

*hos=hospital; pro=probiotic group; con=controlled group; LAB=Lactic acid bacteria; PSB=protected specimen brush; BAL=bronchoalveolar lavage; MV=mechanical ventilation; CFU=colony-forming unit; NT=nasogastric tube; CPIS= clinical pulmonary infection score; EN=enteral nutrition; CRI= New progressive, or persistent infiltration on chest radiograph

Probiotics varied in species and dose in these studies. Lactobacillus rhamnosus GG[14], a multispecies probiotic preparation[15] (containing Lactobacillus casei, L. acidophilus, Bifidobacterium bifidum and mainly L. rhamnosus GG), L. casei rhamnosus[19], Synbiotic 2000FORTE[16,17] acted as the intervention while the doses varied from 2*10 ^9^ CFU to 1*10^11^ on a twic-daily basis. Synbiotic 2000FORTE is a combination of Pediococcus pentosaceus, Leuconostoc mesenteroides, Lactobacillus paracasei subsp paracasei, and Lactobacillus plantarum. Table 2 concluded the outcome data extracted from RCTs included in the Meta-analysis

**Table 2:** Outcome data extracted from RCTs Included in the Meta-analysis.

Study / Year	Group	VAP incidence	Mortality		Length of stay (day)		Duration of MV (day)	Etiology		
			Hospital	Icu	Hospital	Icu		G+	G-	P.A ^d^
Morrow et al/ 2010	Pro	17/68^a^ 13/68^b^	12/68	(-)	21.4±14.9	14.8±11.2	9.5±6.3	10/13	9/13	0/13
	Con	33/70^a^ 28/70^b^	15/70	(-)	21.7±17.4	14.6±11.6	9.6±7.2	15/28	28/28	6/28
Barraud et al/2010	Pro	23/87	21/87	22/87	26.6±22.3	18.7±12.4	(-)	(-)	(-)	(-)
	Con	15/80	21/80	19/80	28.9±26.4	20.2±20.8	(-)	(-)	(-)	(-)
Giamarellos-Bourboulis et al/	Pro	15/36	5/36	5/35	(-)	27.7±15.2	15(5-32)	(-)	(-)	(-)
2009	Con	16/36	10/36	9/30	(-)	41.3±20.5	26(7-60)	(-)	(-)	(-)
Knight et al/2009	Pro	12/130	35/130	28/130	19(8-36)	6(3-11)	5(2-9)	0/12	9/12	0/12
	Con	17/129	42/129	34/129	18(7-32)^c^	7(3-14)^c^	5(3-11)^c^	1/17	11/17	1/17
Forestier et al/2008	Pro	24/102	(-)	(-)	(-)	(-)	(-)	12/24	12/24	3/24
	Con	24/106	(-)	(-)	(-)	(-)	(-)	11/24	13/24	8/24

a: clinically diagnosed VAP. b: microbiologically confirmed VAP. c: Median (interquartile range). d: Pseudomonas aeruginosa

*(-) presents not available

* data are presented as median (range) or mean±SD

*pro=probiotic group; con=controlled group

### 3.3: The Outcomes

#### VAP incidence

**Figure 2:**
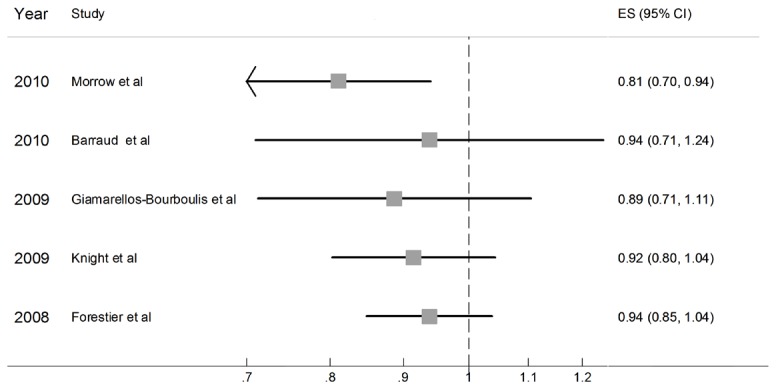
Relative risk for VAP incidence.(D-L).

All these 5 trials reported the incidence of VAP and gained a total number of 844 patients (423 in probiotic group and 421 in placebo group). Figure 2 shows the result of meta-analysis: since heterogeneity was found among the trails (D-L，Q= 12.780 on 4 degrees of freedom, p= 0.012), the D-L random model was chosen. The pooled RR was 0.94 (95%CI 0.85-1.04, p=0.22). Even though probiotic group showed a trend toward lower incidence of VAP, the preventative effect was not statistically valid. But when the Earraud et al or Forestier et al’s trial was excluded respectively, statistically significant conclusion could be drown. 

#### Mortality

**Figure 3:**
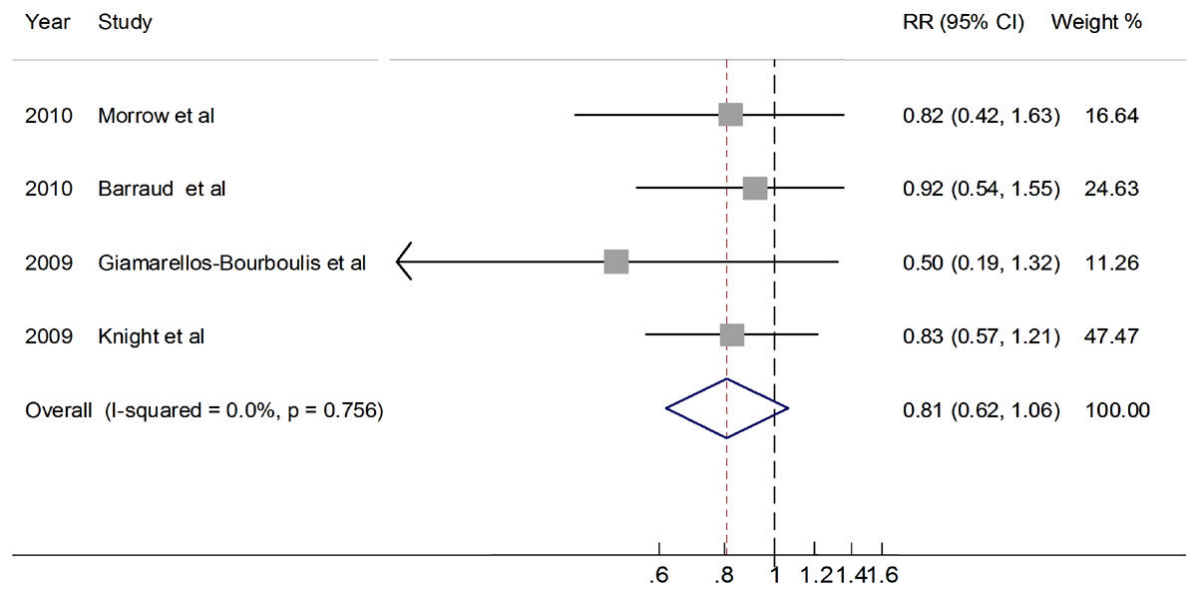
Relative risk for mortality.(M-H).

Four trials provided the data on hospital mortality. Figure 3 shows the result of meta-analysis: Heterogeneity was not found among the comparisons (χ^2^=1.19, p=0.756; I^2^ =0%). There was no difference between patients receiving probiotics and placebo with regard to all cause hospital mortality (636 patients, RR 0.81, 95%CI 0.62-1.06; p=0.13);

Three trials provided the data on lCU mortality. Heterogeneity was not found among the comparisons (p=0.356; I^2^ =3.1%). There was no difference between patients receiving probiotics and placebo with regard to all-cause ICU mortality (491 patients, RR 0.84, 95%CI 0.61-1.16, p=0.29);

#### Length of stay in ICU and in hospital

Three trials provided the data on stay length in ICU. Heterogeneity was found among the comparisons (Q= 6.01, p=0.05). So the random model was chosen. There was no difference between patients receiving probiotics and placebo （356 patients，ES -3.22, 95%CI -9.14,-2.70, p=0.29,)

Less than 2 trials estimated length of stay in hospital, so the outcomes were not analyzed.

#### Etiology studies

To determine the distribution of pathogens responsible for VAP, we also analyzed the ratio of G- and G+ bacteria to the total pathogens. There was no heterogeneity (χ^2^ =1.04, p = 0.59), neither was the incidence of G+ bacterial infection when probiotics was used (118 patients, RR 1.21, 95%CI 0.83-1.75, p=0.33). The risk for G- bacterial infection in VAP patients was not statistically significant either (118 patients, RR 0.87, 95%CI 0.67-1.13, p=0.30).

**Figure 4:**
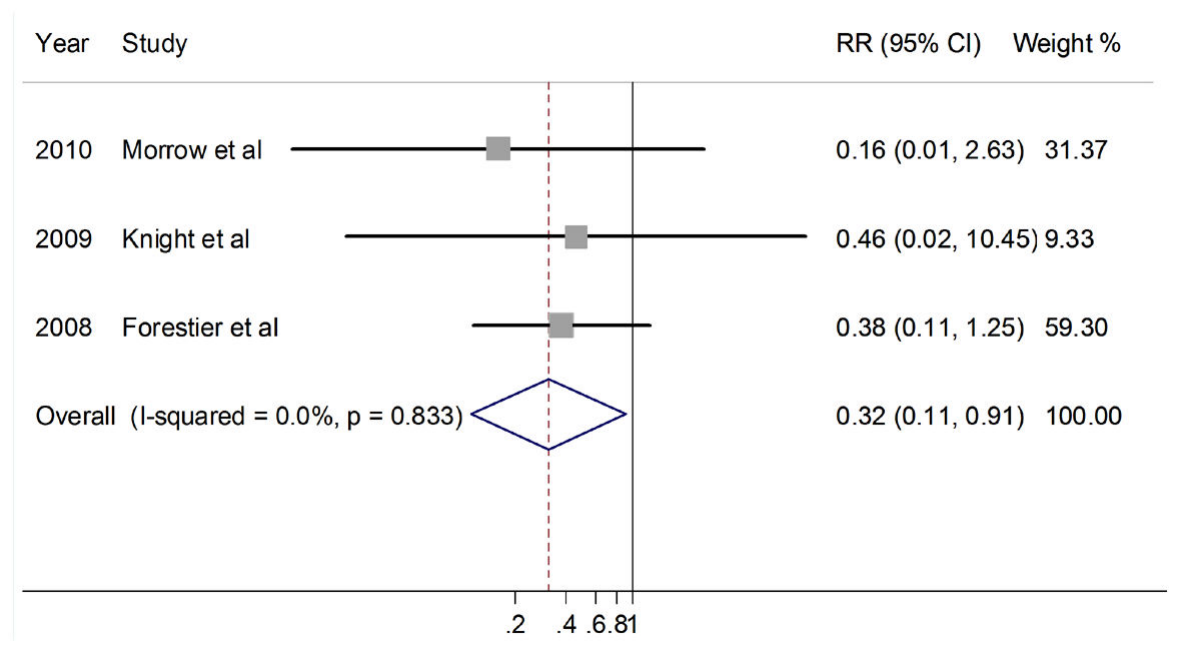
Relative risk for VAP caused by P. aeruginosa.(M-H).

But probiotics did decrease the Pseudomonas aeruginosa infection in VAP. The reduction of relative risk was statistically significant (118 patients, RR 0.36, 95%CI 0.11-0.91, p=0.03) (Figure 4 ). 

The other pathogens such as Acinetobacter baumannii, Enterobacteriaceae, Klebsiella, Yeast were not examined because of the limited data. 

## Discussion

The present meta-analysis showed no statistically significant decrease in incidence of VAP neither the other clinical outcomes such as mortality , length of stay in ICU. A decrease in the P. aeruginosa infection was found. Several points such as heterogeneity, diagnostic criteria, probiotic recipes are critical for comprehensive understand of this topic.

Performing rigorous screening process and statistic work to rule out the methodological influence, we found lack of efficacy of probiotics in preventing ventilator-associated pneumonia, nor were the clinical outcomes. It was calculated that when respectively or simultaneously excluding the studies conducted by Earraud et al[15], Forestier et al[19] from the meta-analysis, positive statistically significant results showed up (RR 0.75, 95%CI 0.58- 0.98, p = 0.03, RR 0.71,95% CI 0.55-0.92, p = 0.01, RR 0.86, 95%CI 0.75-0.999, P = 0.049). These two studies were carried out in comprehensive hospitals where medical patients held a small to moderate percentage of studied patients. This founding rejuvenated enthusiasm to explain. Firstly, statistical significance was marginally lost (the upper limit of confidence interval of only 1.04), which didn’t allow for film conclusion. Secondly, the preventive effect of probiotics was more likely to be established when the populations tend to be trauma, surgical, multi-organ injured patients. It was in accordance to the previous studies advocating that trauma, neurosurgical, liver transplantation patients and general surgical patients could benefit from probiotics in reduction the incidence of pneumonia [30–33]. Oh the other hand, overall RR shifted along with different diagnostic criteria. When the clinical diagnostic criteria was used, the pooled RR was higher than that drawn from microbiologically confirmed diagnosis (RR 0.97, 95%CI 0.92-1.02 for clinical diagnosis and RR 0.94, 95%CI 0.85-1.04 for microbiological diagnosis respectively). Since the situation is delicate, and it seems unrealistic to standardize VAP diagnostic criteria and provide enough RCTs using the same diagnosis criteria, it seems reasonable to rule out the impact of diagnosis and identify the onset of VAP by using unambiguous and subjective outcome indicators. The onset of positive culture, examination or laboratory data and clinical scores might be helpful [34,35].

It has been recognized that the reduction of VAP rates is less important and practical than clinical outcomes such as mortality, the length of stay, the MV duration and antibiotic prescribed. This meta-analysis did not reveal any reduction of ICU or hospital mortality conferred by the administration of probiotics, which was consistent with the previous studies [23,36]. Critically ill patients that although typically presenting with pro-inflammatory conditions(SIRS)，subsequently active compensatory anti-inflammatory response(CARS) characterized by elevated plasma levels of anti-inflammatory cytokines (e.g., IL-10) and a decreased ability of immune cells to respond appropriately to a new inflammatory challenge[37]. Impaired responsiveness of the innate immune system after the onset of critical illness or injury is associated with increased risk of nosocomial infection and death. Under such a delicate and complicated situation, the exact effect of topical administration of ecological preparations is being questioned. Previous studies indicated a change of several inflammatory mediators after probiotics therapy in critically ill patients suffering from multiple injury or severe traumatic brain injury. As to the medical, infected patients, these populations tend to be confronted with complicated clinical status and aggressive antibiotic treatment. On the other hand, the local concentration of probiotics could not provide better efficacy during aggressive antibiotic therapy along with gastric dysmotility.

Probiotic effects can’t be generalized and the characteristics of probiotic strains need to be understood to pair them with specific disease states or desired goals. Specific strains inhibited growth of clinical isolates whereas others superiorly induced the anti-inflammatory cytokine IL-10 and their viability vary in digestive tract environment [38]. The famous study-Probiotics in pancreatitis trail (PROPATRIA)[39]used a mixture of six probiotic strains only two of which have been shown to effectively reduce the growth of pathogens frequently observed in pancreatitis. The mixed recipe is proven better resolution in antimicrobial spectrum, induction of IL-10, and silencing of pro-inflammatory cytokines than the individual components. For instance, the Synbiotic 2000 FORTE has distinguished itself in preventing infections including VAP in trauma, liver transplantation and major abdominal surgery. According to the studies in vitro, the single recipe such as Lactobacillus casei rhamnosus, performed well in inhibiting the potential pathogenic microbes such as Escherichia coli, Enterococcus, Staphylococcus but the tolerance of acid environment was inferior to Bifidobacterium. Hence, the probiotic strains chosen have much to do with its efficacy in preventing VAP and it need to be explored in depth.

According to our study, it sounded promising that probiotics decreased the risk of developing VAP caused by P. aeruginosa. As we explained before, from the very beginning, an increase in antibiotic-resistant pathogens and secondary infections appeared as an inevitable result of antibiotic therapy, and probiotics outperformed antibiotics both in the secondary infection and immunity stimulation. P. aeruginosa, the most common MDR gram-negative bacterial pathogen causing HAP/VAP, has intrinsic resistance to many antimicrobial agents. Previous study[40] indicated that compared with SDD, administration of probiotics demonstrated a superiority in P. aeruginosa prevention and non-inferiority both in Gram-positive cocci and infection prevention. In vitro studies, Lactobacillus fermentum-secreted compound inhibited the growth, cytotoxicity and biofilm formation of several S. aureus and P. aeruginosa strains [41]. Besides, sIC of PLA produced by Lactobacillus probiotic strains, decreased the ability of the tested strains to adhere both to the cellular and inert substrata and induced changes in the adherence patterns as well as in the cell morphology, attenuated the virulence and pathogenicity of P. aeruginosa. In addition, the sIC of PLA induced a significant decrease of sheep red blood cells haemolysins, lecithinase and caseinase and stimulated lipase and gelatinase production by Pseudomonas strains[42]. Apart from the non-specific mechanisms, probiotics exert a preventive effect for VAP by inhibiting colonization of the stomach with the pathogen P.aeruginosa which is intolerant to acid environment. After administration of probiotics, there follows a marked increase in the acetic acid concentration which has an antimicrobial effect on Pseudomonas and Enterobacteriaceae, While Gram-positive cocci (Staphylococcus and Enterococcus) are tolerant to acetic acid. Furthermore, probiotics do not contribute to the development of antibiotic-resistant strains. Once colonized, these strains are genetically stable and not likely to incorporate resistance genes or plasmids or to transfer genetic material, all of which characterize their inherent resistance to certain antibiotics and to other species. 

As mentioned above, previous studies failed to come to an agreement. The previous meta-analysis [24] on the same subject included two studies [18,22] that compared probiotics with CHX or SDD for VAP prevention. It has been proven that oral antiseptic and antibiotics effectively prevent nosocomial infection and VAP [43–45], we figured that they were standard control rather than placebo control. Not only did they compromise the homogeneity of placebo groups, but also underestimate the effectiveness of probiotics and resulted in an invalid conclusion. The latest meta-analysis comprising 10 RCTs (1,218 patients), argued that administration of probiotics reduced the incidence of ICU-acquired pneumonia and was associated with a shorter stay in the ICU [36]. We questioned that in addition to diversity of enrolled populations, the interventional and controlled methods varied greatly. One of the included trials compared parenteral nutrition with enteral supply of fiber and probiotics, one compared synbiotics with prebiotic, and two articles reported repeated results of the same study.. Even though ours missed several trials as they didn’t meet the criteria of high quality, the conclusion was relative robust and inspiring in the MV patients.

**Figure 5:**
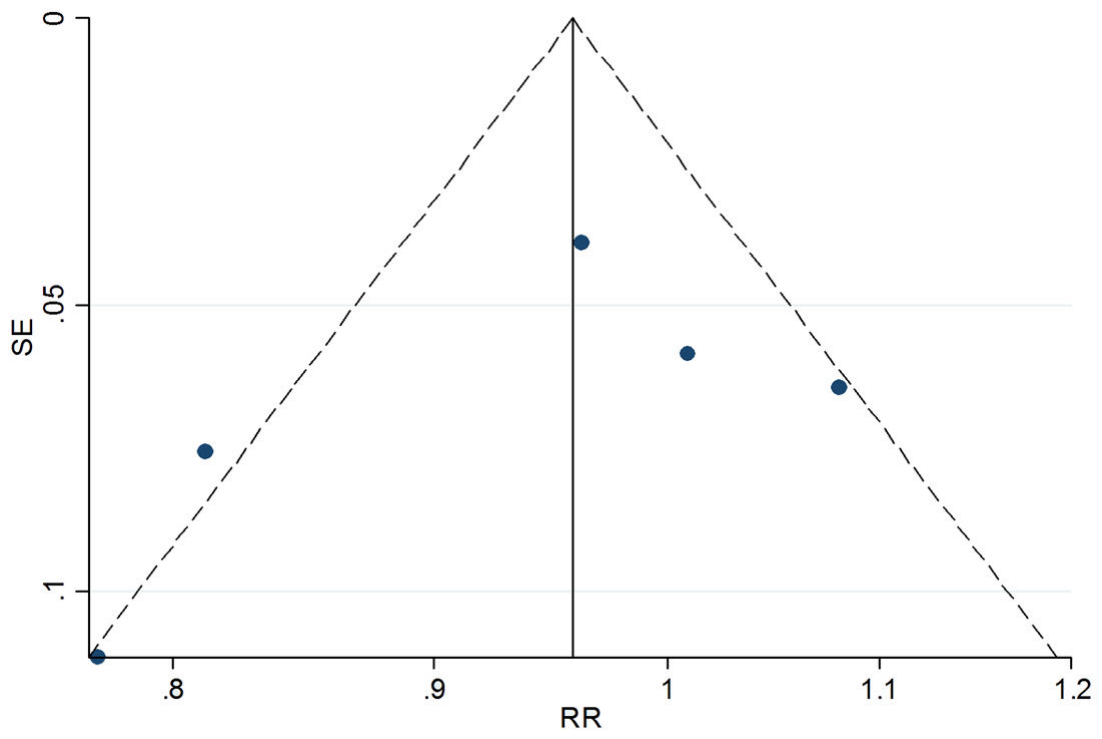
Funnel plot with pseudo 95% confidence limits. *RR=relative risk; SE=standard error.

Besides, our meta-analysis was confronted with several shortcomings. 1. Since there were few qualified RCTs, we fell short of our hope to unite the diagnostic criteria of VAP and probiotics The biases have been discussed above. 2. Heterogeneity was found among trials (D-l model, P=0.01), in other words, the diversity between studies was too obvious to be ignored. Such diversity is commonly referred to as methodological or clinical heterogeneity, and may or may not be responsible for observed discrepancies in the results of the studies. As to our meta-analysis, the population, probiotic used and diagnosis criteria of VAP bore the primary responsibility for heterogeneity. 3. In addition, although we extensively searched for relevant studies using multiple databases and multiple search items, and no language restriction was placed on the search, a funnel plot suggested the possibility of publication bias (Figure 5).4 The conclusion was drawn from highly selected population and the external validation was questionable, so it had trouble in application to expanded populations. 

All over, the probiotic prophylaxis of ventilator-associated pneumonia was still uncertain but did decrease the ventilator-associated pneumonia caused by P. aeruginosa. It is recommended that probiotics be applied to specific population and more high-quality RCTs be carried out.

## Supporting Information

Checklist S1 **PRISMA checklist.**
(DOC)
